# 3,4,7,8-Tetra­methyl-1,10-phenanthrolin-1-ium nitrate monohydrate

**DOI:** 10.1107/S1600536812023318

**Published:** 2012-05-31

**Authors:** Ke-Jie Zhang, Yan-Fang Zhang

**Affiliations:** a School of Material Science and Engineering, Nanjing Institute of Science and Technology, Nanjing 211168, People’s Republic of China; b Department of Life Science and Technology, Xinxiang College, Xinxiang 453003, People’s Republic of China

## Abstract

In the crystal of the title compound, C_16_H_17_N_2_^+^·NO_3_^−^·H_2_O, the tetra­methyl-1,10-phenanthrolinium cations, nitrate anions and lattice water mol­ecules are all located on a mirror plane with the methyl H atoms of the cation equally disordered over two sites about the mirror plane. The cation, anion and water mol­ecule are linked by O—H⋯O and N—H⋯O hydrogen bonds into a sheet parallel to the *bc* plane. π–π stacking between phenanthroline ring systems is observed in the crystal structure, the centroid–centroid distance being 3.4745 (6) Å.

## Related literature

For proton-transfer structures of phenanthroline and its derivatives, see: Bei *et al.* (2004 ▶); Buttery *et al.* (2006 ▶); Gillard *et al.* (1998 ▶); Harvey *et al.* (2008 ▶); Hensen *et al.* (1998 ▶, 2000 ▶); Kolev *et al.* (2009 ▶); Lin *et al.* (2009 ▶); Maresca *et al.* (1989 ▶); Milani *et al.* (1997 ▶); Montagu-Bourin *et al.* (1981 ▶); Shang *et al.* (2006 ▶); Thevenet & Rodier (1978 ▶); Thevenet *et al.* (1977 ▶, 1978 ▶, 1980 ▶); Wang *et al.* (1999 ▶); Yu *et al.* (2006 ▶).
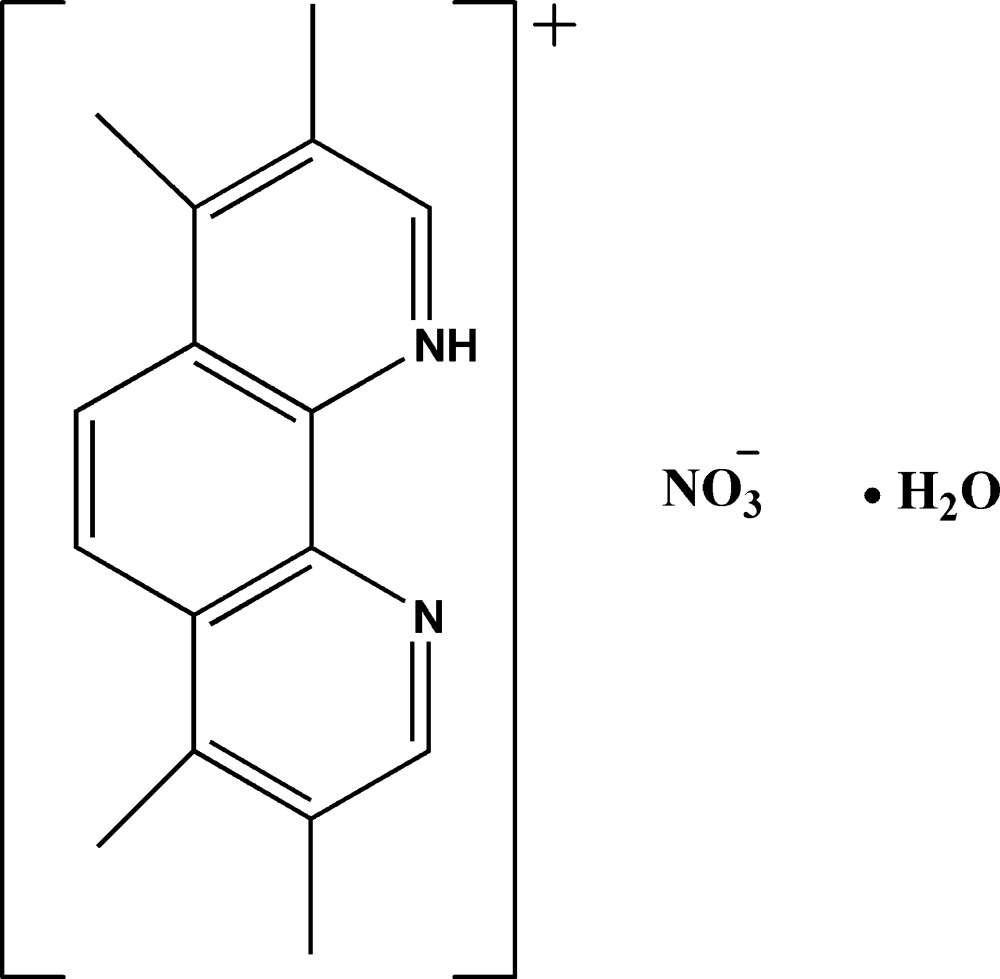


## Experimental

### 

#### Crystal data

C_16_H_17_N_2_^+^·NO_3_^−^·H_2_O
*M* *_r_* = 317.34
Orthorhombic, 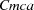

*a* = 6.7401 (8) Å
*b* = 24.090 (3) Å
*c* = 19.254 (2) Å
*V* = 3126.1 (6) Å^3^
*Z* = 8
Mo *K*α radiation
μ = 0.10 mm^−1^
*T* = 296 K
0.37 × 0.30 × 0.21 mm

#### Data collection

Bruker SMART 1000 CCD area-detector diffractometer
11308 measured reflections
1585 independent reflections
1149 reflections with *I* > 2σ(*I*)
*R*_int_ = 0.029

#### Refinement

*R*[*F* ^2^ > 2σ(*F* ^2^)] = 0.052
*wR*(*F* ^2^) = 0.153
*S* = 1.03
1585 reflections
143 parameters
H-atom parameters constrained
Δρ_max_ = 0.22 e Å^−3^
Δρ_min_ = −0.27 e Å^−3^

### 

Data collection: *SMART* (Bruker, 1997 ▶); cell refinement: *SAINT* (Bruker, 1997 ▶); data reduction: *SAINT*; program(s) used to solve structure: *SHELXTL* (Sheldrick, 2008 ▶); program(s) used to refine structure: *SHELXTL*; molecular graphics: *SHELXTL*; software used to prepare material for publication: *publCIF* (Westrip, 2010 ▶).

## Supplementary Material

Crystal structure: contains datablock(s) I, global. DOI: 10.1107/S1600536812023318/xu5537sup1.cif

Structure factors: contains datablock(s) I. DOI: 10.1107/S1600536812023318/xu5537Isup2.hkl

Supplementary material file. DOI: 10.1107/S1600536812023318/xu5537Isup3.cml

Additional supplementary materials:  crystallographic information; 3D view; checkCIF report

## Figures and Tables

**Table 1:** Hydrogen-bond geometry (Å, °)

*D*—H⋯*A*	*D*—H	H⋯*A*	*D*⋯*A*	*D*—H⋯*A*
N2—H2⋯O4^i^	0.86	1.86	2.692 (3)	164
O4—H1*W*⋯O1^ii^	0.84	1.97	2.808 (4)	174
O4—H2*W*⋯O1^iii^	0.83	2.07	2.886 (4)	170

Symmetry codes: (i) 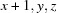; (ii) 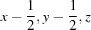; (iii) 
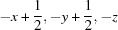
.

**Fig. 1.**
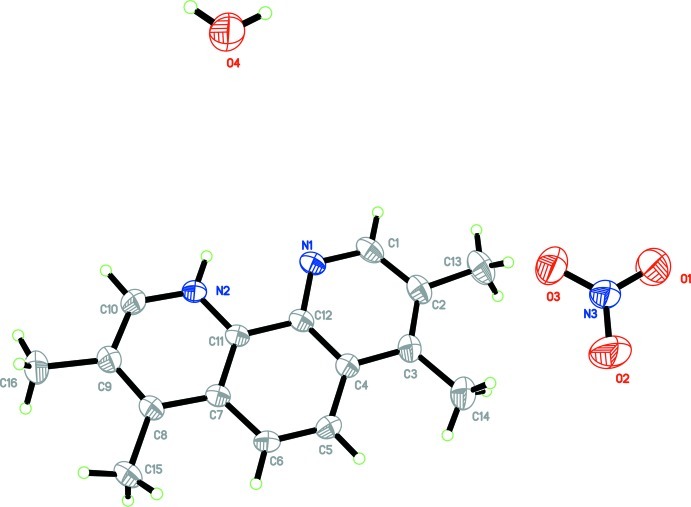
The molecular structure of the title compound, with atom labels and 30% probability displacement ellipsoids for non-H atoms.

**Fig. 2.**
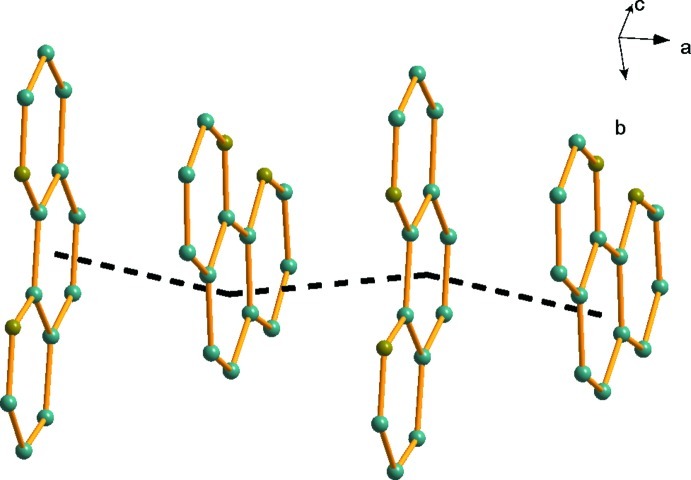
π-π interactions between the neighboring aromatic rings of the title compound. Aromatic hydrogen atoms and methyl groups have been omitted for clarity.

## supplementary crystallographic information

### Comment

1,10-Phenanthroline and its derivatives have been recognized as good proton acceptors, and usually are considered a suitable agent in the synthesis of proton-transfer systems. Some proton-transfer complexes based on 1,10-phenanthroline (Buttery *et al.* 2006; Gillard *et al.* 1998; Hensen *et al.* 1998, 2000; Kolev *et al.* 2009; Maresca *et al.* 1989; Milani *et al.* 1997; Montagu-Bourin, Levillain, Ceolin, Thevenet & Souleau 1981; Shang *et al.* 2006; Thevenet & Rodier 1978; Thevenet *et al.* 1977, 1980; Thevenet, Rodier & Khodadad 1978; Wang *et al.* 1999), 2,9-dimethyl-1,10-phenanthroline (Harvey *et al.* 2008; Yu *et al.* 2006), and 6-nitro-1,10-phenanthroline (Bei *et al.* 2004), 5,6-dihydroxy-phenanthroline (Lin *et al.* 2009) have been synthesized. In the recent work, the title compound (I), C_16_H_17_N_2_]NO_3_.H_2_O, was obtained unintentionally as a major product in the reaction of Tb(NO_3_)_3_.6H_2_O with the 3,4,7,8-tetramethyl-1,10-phenanthroline in water. To the best our knowledge, this is the first example of proton-transfer system containing 3,4,7,8-tetramethyl-1,10- 1,10-phenanthroline.

The numbering scheme of (I) is given in Fig. 1, and the selected bond lengths and bond angles are provided in the cif file. The crystal contains one protonated 3,4,7,8-tetramethyl-1,10- 1,10-phenanthroline cation, one nitrate anion and one water molecule. In the crystal structure, the cations, anions and water molecules are linked into two dimensional layers parallel to the *bc* plane by N—H···O and O—H···O hydrogen bonds (Table 1). Among them, N—H···O hydrogen bonds play a very important role in the formation of proton-transfer compounds. Additionally, the monoprotonated 3,4,7,8-tetramethyl-1,10- 1,10-phenanthroline cations are parallel to each other in the crystal packing, showing π-π interactions (Fig. 2); the centroid–centroid distance is 3.4745 (6) Å.

### Experimental

A aqueous solution (12 ml) of Tb(NO_3_)_3_.6H_2_O (1 mmol) and 3,4,7,8-tetramethyl-1,10-phenanthroline (1 mmol) was stirred. The mixture was then transferred to a 25-ml Teflon reactor and kept at 433 K for 3 d under autogenous pressure, and then cooled to room temperature at a rate of 10 K h^-1^. Colorless crystals of the title compound were obtained.

### Refinement

The carbon-bound H atoms were placed in calculated positions and were included in the refinement in the riding model approximation, with C—H = 0.93 Å, *U_iso_*(H) = 1.2*U_eq_*(C aromatic) and C—H = 0.96 Å, *U_iso_*(H) = 1.5*U_eq_*(C methyl), respectively. The H atoms bound to O were located in a difference Fourier map, and refined as riding in their as-found relative positions with *U_iso_*(H) = 1.5*U_eq_*(O). The methyl H atoms are equally disordered over two sites about the mirror plane.

### Crystal data

**Table:**

C_16_H_17_N_2_^+^·NO_3_^−^·H_2_O	*F*(000) = 1344
*M**_r_* = 317.34	*D*_x_ = 1.349 Mg m^−^^3^
Orthorhombic, *C**m**c**a*	Mo *K*α radiation, λ = 0.71073 Å
Hall symbol: -C 2bc 2	Cell parameters from 2972 reflections
*a* = 6.7401 (8) Å	θ = 2.7–25.0°
*b* = 24.090 (3) Å	µ = 0.10 mm^−^^1^
*c* = 19.254 (2) Å	*T* = 296 K
*V* = 3126.1 (6) Å^3^	Block, colorless
*Z* = 8	0.37 × 0.30 × 0.21 mm

### Data collection

**Table:**

Bruker SMART 1000 CCD area-detector diffractometer	1149 reflections with *I* > 2σ(*I*)
Radiation source: fine-focus sealed tube	*R*_int_ = 0.029
Graphite monochromator	θ_max_ = 25.5°, θ_min_ = 2.7°
φ and ω scans	*h* = −8→7
11308 measured reflections	*k* = −28→28
1585 independent reflections	*l* = −23→23

### Refinement

**Table:**

Refinement on *F*^2^	Primary atom site location: structure-invariant direct methods
Least-squares matrix: full	Secondary atom site location: difference Fourier map
*R*[*F*^2^ > 2σ(*F*^2^)] = 0.052	Hydrogen site location: inferred from neighbouring sites
*wR*(*F*^2^) = 0.153	H-atom parameters constrained
*S* = 1.03	*w* = 1/[σ^2^(*F*_o_^2^) + (0.0707*P*)^2^ + 2.7655*P*] where *P* = (*F*_o_^2^ + 2*F*_c_^2^)/3
1585 reflections	(Δ/σ)_max_ < 0.001
143 parameters	Δρ_max_ = 0.22 e Å^−^^3^
0 restraints	Δρ_min_ = −0.27 e Å^−^^3^

### Special details

**Table:**

Geometry. All e.s.d.'s (except the e.s.d. in the dihedral angle between two l.s. planes) are estimated using the full covariance matrix. The cell e.s.d.'s are taken into account individually in the estimation of e.s.d.'s in distances, angles and torsion angles; correlations between e.s.d.'s in cell parameters are only used when they are defined by crystal symmetry. An approximate (isotropic) treatment of cell e.s.d.'s is used for estimating e.s.d.'s involving l.s. planes.
Refinement. Refinement of *F*^2^ against ALL reflections. The weighted *R*-factor *wR* and goodness of fit *S* are based on *F*^2^, conventional *R*-factors *R* are based on *F*, with *F* set to zero for negative *F*^2^. The threshold expression of *F*^2^ > σ(*F*^2^) is used only for calculating *R*-factors(gt) *etc*. and is not relevant to the choice of reflections for refinement. *R*-factors based on *F*^2^ are statistically about twice as large as those based on *F*, and *R*- factors based on ALL data will be even larger.

### Fractional atomic coordinates and isotropic or equivalent isotropic displacement parameters (Å^2^)

**Table:**

	*x*	*y*	*z*	*U*_iso_*/*U*_eq_	Occ. (<1)
C1	1.0000	0.19047 (14)	0.08694 (15)	0.0642 (9)	
H1	1.0000	0.1825	0.0397	0.077*	
C2	1.0000	0.24624 (14)	0.10669 (16)	0.0618 (8)	
C3	1.0000	0.25954 (12)	0.17669 (16)	0.0550 (8)	
C4	1.0000	0.21518 (11)	0.22551 (14)	0.0474 (7)	
C5	1.0000	0.22191 (12)	0.29933 (15)	0.0517 (7)	
H5	1.0000	0.2576	0.3177	0.062*	
C6	1.0000	0.17823 (11)	0.34314 (14)	0.0520 (7)	
H6	1.0000	0.1847	0.3908	0.062*	
C7	1.0000	0.12218 (11)	0.31849 (13)	0.0485 (7)	
C8	1.0000	0.07458 (12)	0.36186 (14)	0.0561 (8)	
C9	1.0000	0.02213 (12)	0.33180 (16)	0.0635 (9)	
C10	1.0000	0.01823 (12)	0.26008 (17)	0.0660 (9)	
H10	1.0000	−0.0168	0.2396	0.079*	
C11	1.0000	0.11486 (10)	0.24627 (14)	0.0483 (7)	
C12	1.0000	0.16081 (12)	0.19900 (13)	0.0485 (7)	
C13	1.0000	0.29005 (16)	0.05051 (19)	0.0890 (12)	
H13A	1.0207	0.2728	0.0062	0.134*	0.50
H13B	1.1046	0.3162	0.0592	0.134*	0.50
H13C	0.8747	0.3090	0.0505	0.134*	0.50
C14	1.0000	0.31938 (13)	0.1996 (2)	0.0764 (10)	
H14A	0.8712	0.3352	0.1918	0.115*	0.50
H14B	1.0972	0.3397	0.1734	0.115*	0.50
H14C	1.0316	0.3214	0.2481	0.115*	0.50
C15	1.0000	0.08170 (15)	0.43955 (15)	0.0770 (11)	
H15A	0.9482	0.0488	0.4610	0.116*	0.50
H15B	0.9186	0.1129	0.4518	0.116*	0.50
H15C	1.1332	0.0880	0.4554	0.116*	0.50
C16	1.0000	−0.03134 (13)	0.3737 (2)	0.0912 (13)	
H16A	1.0869	−0.0274	0.4129	0.137*	0.50
H16B	1.0453	−0.0614	0.3450	0.137*	0.50
H16C	0.8679	−0.0390	0.3896	0.137*	0.50
N1	1.0000	0.14820 (10)	0.13060 (11)	0.0570 (7)	
N2	1.0000	0.06277 (9)	0.21952 (11)	0.0574 (7)	
H2	1.0000	0.0585	0.1752	0.069*	
N3	0.5000	0.38702 (12)	0.09781 (14)	0.0702 (8)	
O1	0.5000	0.41620 (12)	0.04627 (14)	0.1338 (15)	
O2	0.5000	0.40810 (14)	0.15514 (15)	0.1348 (15)	
O3	0.5000	0.33707 (11)	0.09262 (15)	0.1115 (11)	
O4	0.0000	0.02821 (10)	0.08654 (12)	0.1174 (13)	
H2W	0.0000	0.0477	0.0512	0.176*	
H1W	0.0000	−0.0058	0.0779	0.176*	

### Atomic displacement parameters (Å^2^)

**Table:**

	*U*^11^	*U*^22^	*U*^33^	*U*^12^	*U*^13^	*U*^23^
C1	0.080 (2)	0.077 (2)	0.0361 (15)	0.000	0.000	0.0103 (14)
C2	0.066 (2)	0.067 (2)	0.0532 (18)	0.000	0.000	0.0177 (15)
C3	0.0574 (18)	0.0486 (16)	0.0589 (18)	0.000	0.000	0.0098 (13)
C4	0.0515 (16)	0.0464 (15)	0.0444 (15)	0.000	0.000	0.0003 (11)
C5	0.0607 (18)	0.0442 (15)	0.0501 (16)	0.000	0.000	−0.0088 (12)
C6	0.0679 (19)	0.0516 (16)	0.0365 (14)	0.000	0.000	−0.0074 (12)
C7	0.0616 (18)	0.0480 (15)	0.0360 (13)	0.000	0.000	−0.0022 (11)
C8	0.078 (2)	0.0541 (17)	0.0366 (14)	0.000	0.000	0.0036 (12)
C9	0.093 (2)	0.0498 (17)	0.0477 (17)	0.000	0.000	0.0039 (13)
C10	0.101 (3)	0.0448 (16)	0.0521 (18)	0.000	0.000	−0.0060 (13)
C11	0.0632 (18)	0.0475 (15)	0.0343 (13)	0.000	0.000	−0.0024 (11)
C12	0.0574 (17)	0.0528 (16)	0.0354 (13)	0.000	0.000	0.0007 (11)
C13	0.110 (3)	0.091 (3)	0.067 (2)	0.000	0.000	0.036 (2)
C14	0.091 (3)	0.055 (2)	0.083 (2)	0.000	0.000	0.0130 (17)
C15	0.125 (3)	0.070 (2)	0.0363 (15)	0.000	0.000	0.0060 (14)
C16	0.149 (4)	0.053 (2)	0.072 (2)	0.000	0.000	0.0145 (17)
N1	0.0772 (17)	0.0600 (14)	0.0338 (11)	0.000	0.000	0.0002 (10)
N2	0.0918 (19)	0.0467 (13)	0.0336 (11)	0.000	0.000	−0.0057 (10)
N3	0.095 (2)	0.0645 (18)	0.0508 (16)	0.000	0.000	−0.0033 (13)
O1	0.259 (5)	0.0823 (19)	0.0605 (16)	0.000	0.000	0.0153 (15)
O2	0.233 (4)	0.104 (2)	0.0669 (18)	0.000	0.000	−0.0228 (17)
O3	0.178 (3)	0.0621 (17)	0.095 (2)	0.000	0.000	0.0016 (15)
O4	0.238 (4)	0.0661 (15)	0.0483 (14)	0.000	0.000	−0.0128 (11)

### Geometric parameters (Å, º)

**Table:**

C1—N1	1.320 (4)	C11—N2	1.356 (3)
C1—C2	1.396 (5)	C11—C12	1.433 (4)
C1—H1	0.9300	C12—N1	1.351 (3)
C2—C3	1.385 (4)	C13—H13A	0.9600
C2—C13	1.511 (4)	C13—H13B	0.9600
C3—C4	1.423 (4)	C13—H13C	0.9600
C3—C14	1.508 (4)	C14—H14A	0.9600
C4—C12	1.406 (4)	C14—H14B	0.9600
C4—C5	1.431 (4)	C14—H14C	0.9600
C5—C6	1.349 (4)	C15—H15A	0.9600
C5—H5	0.9300	C15—H15B	0.9600
C6—C7	1.431 (4)	C15—H15C	0.9600
C6—H6	0.9300	C16—H16A	0.9600
C7—C11	1.402 (4)	C16—H16B	0.9600
C7—C8	1.419 (4)	C16—H16C	0.9600
C8—C9	1.390 (4)	N2—H2	0.8600
C8—C15	1.506 (4)	N3—O3	1.208 (4)
C9—C10	1.384 (5)	N3—O2	1.215 (4)
C9—C16	1.520 (4)	N3—O1	1.216 (4)
C10—N2	1.327 (4)	O4—H2W	0.8276
C10—H10	0.9300	O4—H1W	0.8365
			
N1—C1—C2	124.7 (3)	N1—C12—C11	116.4 (2)
N1—C1—H1	117.7	C4—C12—C11	119.3 (2)
C2—C1—H1	117.7	C2—C13—H13A	109.5
C3—C2—C1	119.2 (3)	C2—C13—H13B	109.5
C3—C2—C13	122.3 (3)	H13A—C13—H13B	109.5
C1—C2—C13	118.5 (3)	C2—C13—H13C	109.5
C2—C3—C4	118.0 (3)	H13A—C13—H13C	109.5
C2—C3—C14	120.4 (3)	H13B—C13—H13C	109.5
C4—C3—C14	121.7 (3)	C3—C14—H14A	109.5
C12—C4—C3	117.4 (2)	C3—C14—H14B	109.5
C12—C4—C5	117.8 (2)	H14A—C14—H14B	109.5
C3—C4—C5	124.8 (3)	C3—C14—H14C	109.5
C6—C5—C4	122.2 (3)	H14A—C14—H14C	109.5
C6—C5—H5	118.9	H14B—C14—H14C	109.5
C4—C5—H5	118.9	C8—C15—H15A	109.5
C5—C6—C7	121.9 (2)	C8—C15—H15B	109.5
C5—C6—H6	119.0	H15A—C15—H15B	109.5
C7—C6—H6	119.0	C8—C15—H15C	109.5
C11—C7—C8	118.8 (2)	H15A—C15—H15C	109.5
C11—C7—C6	116.6 (2)	H15B—C15—H15C	109.5
C8—C7—C6	124.6 (2)	C9—C16—H16A	109.5
C9—C8—C7	119.3 (2)	C9—C16—H16B	109.5
C9—C8—C15	121.2 (3)	H16A—C16—H16B	109.5
C7—C8—C15	119.5 (3)	C9—C16—H16C	109.5
C10—C9—C8	118.5 (3)	H16A—C16—H16C	109.5
C10—C9—C16	118.2 (3)	H16B—C16—H16C	109.5
C8—C9—C16	123.3 (3)	C1—N1—C12	116.5 (3)
N2—C10—C9	122.2 (3)	C10—N2—C11	121.6 (2)
N2—C10—H10	118.9	C10—N2—H2	119.2
C9—C10—H10	118.9	C11—N2—H2	119.2
N2—C11—C7	119.5 (2)	O3—N3—O2	119.4 (3)
N2—C11—C12	118.3 (2)	O3—N3—O1	120.6 (3)
C7—C11—C12	122.2 (2)	O2—N3—O1	120.0 (3)
N1—C12—C4	124.3 (2)	H2W—O4—H1W	113.2
			
N1—C1—C2—C3	0.0	C15—C8—C9—C16	0.0
N1—C1—C2—C13	180.0	C8—C9—C10—N2	0.0
C1—C2—C3—C4	0.0	C16—C9—C10—N2	180.0
C13—C2—C3—C4	180.0	C8—C7—C11—N2	0.0
C1—C2—C3—C14	180.0	C6—C7—C11—N2	180.0
C13—C2—C3—C14	0.0	C8—C7—C11—C12	180.0
C2—C3—C4—C12	0.0	C6—C7—C11—C12	0.0
C14—C3—C4—C12	180.0	C3—C4—C12—N1	0.0
C2—C3—C4—C5	180.0	C5—C4—C12—N1	180.0
C14—C3—C4—C5	0.0	C3—C4—C12—C11	180.0
C12—C4—C5—C6	0.0	C5—C4—C12—C11	0.0
C3—C4—C5—C6	180.0	N2—C11—C12—N1	0.0
C4—C5—C6—C7	0.0	C7—C11—C12—N1	180.0
C5—C6—C7—C11	0.0	N2—C11—C12—C4	180.0
C5—C6—C7—C8	180.0	C7—C11—C12—C4	0.0
C11—C7—C8—C9	0.0	C2—C1—N1—C12	0.0
C6—C7—C8—C9	180.0	C4—C12—N1—C1	0.0
C11—C7—C8—C15	180.0	C11—C12—N1—C1	180.0
C6—C7—C8—C15	0.0	C9—C10—N2—C11	0.0
C7—C8—C9—C10	0.0	C7—C11—N2—C10	0.0
C15—C8—C9—C10	180.0	C12—C11—N2—C10	180.0
C7—C8—C9—C16	180.0		

### Hydrogen-bond geometry (Å, º)

**Table:**

*D*—H···*A*	*D*—H	H···*A*	*D*···*A*	*D*—H···*A*
N2—H2···O4^i^	0.86	1.86	2.692 (3)	164
O4—H1*W*···O1^ii^	0.84	1.97	2.808 (4)	174
O4—H2*W*···O1^iii^	0.83	2.07	2.886 (4)	170

Symmetry codes: (i) *x*+1, *y*, *z*; (ii) *x*−1/2, *y*−1/2, *z*; (iii) −*x*+1/2, −*y*+1/2, −*z*.
